# Characteristics associated with SARS-CoV-2 testing, infection and vaccine uptake among essential non-healthcare workers in Montréal, 2021

**DOI:** 10.14745/ccdr.v50i06a05

**Published:** 2024-06-28

**Authors:** Chelsea Caya, Dick Menzies, Jesse Papenburg, Cedric Yansouni, Jonathon Campbell

**Affiliations:** 1 Research Institute of the McGill University Health Centre, Montréal, QC; 2 McGill Interdisciplinary Initiative in Infection and Immunity, Montréal, QC; 3 Respiratory Epidemiology and Clinical Research Unit, Centre for Outcomes Research and Evaluation, Research Institute of the McGill University Health Centre, Montréal, QC; 4 McGill International TB Centre, Montréal, QC; 5 Department of Epidemiology, Biostatistics and Occupational Health, Faculty of Medicine and Health Sciences, McGill University, Montréal, QC; 6 Division of Pediatric Infectious Diseases, Department of Pediatrics, Montreal Children’s Hospital, Montréal, QC; 7 Divisions of Infectious Diseases and Medical Microbiology, McGill University Health Centre, Montréal, QC; 8 J.D. MacLean Centre for Tropical Diseases, McGill University, Montréal, QC; 9 Departments of Medicine & Global and Public Health, Faculty of Medicine and Health Sciences, McGill University, Montréal, QC

**Keywords:** SARS-CoV-2, vaccination, public health, infectious disease

## Abstract

**Background:**

Essential non-healthcare workers experienced higher rates of SARS-CoV-2 infection compared to non-essential workers.

**Objective:**

Identify characteristics associated with SARS-CoV-2 testing, infection and vaccine uptake among essential non-healthcare workers in Montréal, Québec.

**Methods:**

Secondary, cross-sectional analysis of data collected from participants prospectively recruited in two observational studies (first study, Onsite Testing Study, January–March 2021; second study, Self-Testing Study, July–October 2021) of essential non-healthcare workers in 2021. Logistic regression with generalized linear mixed models was used to explore characteristics associated with our outcomes (previous SARS-CoV-2 testing, exposure and vaccination).

**Results:**

Overall, 2,755 participants were included (first study, Onsite Testing Study, n=2,128; and second study, Self-Testing Study, n=627). A higher proportion of participants identified as male (n=1,601; 58%), non-White (n=1,527; 55%) and worked in the manufacturing/supplier sector (n=1,706; 62%). Relative to the first study, Onsite Testing Study, participants in the second study, Self-Testing Study, had higher odds (78% vs. 46%; aOR 4.1, 95% CI: 3.2–5.2) of previous SARS-CoV-2 testing and of testing positive prior to study enrolment (6.2% vs. 4.3%; aOR 1.7, 95% CI: 1.1–2.6). Individuals reporting recent SARS-CoV-2 exposure had higher odds of previous SARS-CoV-2 testing (aOR 4.0, 95% CI: 3.0–5.4), while older age (aOR 0.98, 95% CI: 0.98–0.99 per one-year increase) and being male (aOR 0.6, 95% CI: 0.5–0.7) were associated with lower odds of previous testing. Results were similar in stratified analyses. Participants from businesses with more than 50 employees had higher odds of having received a SARS-CoV-2 vaccine (91% vs. 80%; aOR 2.6, 95% CI: 1.4–4.8).

**Conclusion:**

Consideration of individual and business characteristics associated with testing and vaccination programs for SARS-CoV-2 could improve equity, uptake and impact.

## Introduction

The SARS-CoV-2 transmission continues globally (1). Certain populations have been differentially impacted by SARS-CoV-2, such as visible minorities and those with jobs considered “high risk” (2). Notably, essential non-healthcare workers working in-person have experienced higher rates of SARS-CoV-2 infection compared to non-essential workers and those who were able to work from home (3–6). In Montréal, Canada, essential non-healthcare workplaces were most commonly implicated in large outbreaks (7).

In 2021, we conducted two studies among non-healthcare essential workers in Montréal, Canada. In the first study, we visited businesses to assess onsite sampling for SARS-CoV-2 testing from January to March 2021 (8); a period with substantial public health measures to curb SARS-CoV-2 transmission and prior to the wide availability of SARS-CoV-2 vaccines. In the second study, we evaluated self-testing for SARS-CoV-2 with rapid diagnostic tests in similar businesses, from July to October 2021 (9); a period with minimal public health measures in effect and after all adults were eligible for SARS-CoV-2 vaccination (10). These studies, conducted among similar populations during periods with differential public health measures and vaccine availability, provide an opportunity to better understand characteristics associated with SARS-CoV-2 testing and infection, vaccine uptake and population behaviours.

The aim of this study was to leverage data collected during two prospective studies among individuals from non-healthcare businesses in Montréal in 2021, to conduct descriptive, exploratory analyses to identify characteristics associated with SARS-CoV-2 testing and infection, vaccine uptake, and population behaviours (e.g., travel outside Montréal and the province of Québec).

## Methods

### Study designs, participants and procedures

We conducted a secondary analysis of data collected from participants prospectively recruited in two studies. The first (hereafter, the “Onsite Testing Study”) was a prospective, cross-sectional study taking place from January 27 to March 12, 2021, and the second (hereafter, the “Self-Testing Study”) was a prospective, cross-sectional study from July 7 to October 8, 2021. Identical questionnaires were used in both studies except for additional questions related to vaccination in the Self-Testing Study, **Appendix 1**, **Supplemental material**. Detailed descriptions of the individual studies are available elsewhere (8,9).

### Onsite Testing Study

In this study, non-healthcare essential businesses primarily within the borough of Montréal-Nord were contacted. Businesses could be of any size and eligible employees were those 18 years of age and older who were asymptomatic and who had not tested positive for SARS-CoV-2 in the previous four weeks. Our study team visited participating businesses to collect saline gargle samples for SARS-CoV-2 testing from consenting employees present on the day of our visit.

### Self-Testing Study

In this study, non-healthcare businesses in the Greater Montréal Area identified by Montréal Public Health as having at least two cases of SARS-CoV-2 within the last 14 days were contacted. Participant eligibility was identical to the Onsite Testing Study. However we preferentially visited businesses with more than 50 employees. At participating businesses, consenting employees present on the day of our visit performed a SARS-CoV-2 rapid antigen detection test (Panbio^TM^ COVID-19 Ag Rapid Test Device; Abbott Laboratories) under the supervision of the study team.

Public health measures and vaccine availability in Montréal during 2021

Public health measures and vaccine availability differed between included studies, with extensive public health measures and travel restrictions within Québec and Canada in place during the Onsite Testing Study period, and comparatively fewer measures and interprovincial travel restrictions in effect during the Self-Testing Study period.

Throughout Québec, a province-wide curfew from 8:00 p.m. to 5:00 a.m. was instituted on January 9, 2021, ending on May 28, 2021 ((11,12)), encompassing the entirety of the Onsite Testing Study period. With respect to travel limitations, non-essential travel was discouraged until May 28, 2021, while the border between Ontario and Québec was closed for non-essential travel from April 19 to June 16, 2021 ((13,14)). Public health measures also included the closing of all non-essential businesses in Montréal from December 25, 2020, until February 8, 2021 ((15,16)). Gradually and up to June 28, 2021, most public health measures were relaxed. Gathering and capacity limits, however, remained in place ((17)) and were increased on August 1, 2021. No additional public health measures were imposed until December 16, 2021, due to the Omicron variant ((18,19)).

The rollout of SARS-CoV-2 vaccines in Montréal began on March 1 and by May 14, 2021, all adults in Québec were eligible to receive a SARS-CoV-2 vaccine, approximately 10 weeks prior to the start of the Self-Testing Study ((10,20)).

### Statistical analyses

We performed descriptive analyses using medians and interquartile ranges (IQR) for continuous data and proportions for categorical data for individual and business characteristics of the total population, as well as for each study population separately. Characteristics between the two study populations were compared using appropriate statistical tests (i.e., Kruskal-Wallis tests for continuous variables, and chi-squared or Fisher’s exact tests for categorical variables).

Individual characteristics evaluated included age (continuous), sex (male, female), self-reported ethnicity (White, non-White), household income based on forward sortation area (top 60% income quintile, bottom 40%), self-reported presence of a health condition (yes, no) and self-reported smoking history (never, current/previous smoker). Business characteristics included sector (manufacturer/supplier, retail/consumer facing, office, childcare) and business size (50 employees or fewer, more than 50 employees). Questionnaires and data harmonization between studies are described in **Appendix 2**, **Supplemental material**, **Tables S1 to S2**, respectively.

We evaluated five outcomes: 1) receipt of a SARS-CoV-2 test prior to study enrolment; 2) positive SARS-CoV-2 test result more than four weeks prior to study enrolment; 3) self-reported travel outside the Montréal area or Québec in the previous 14 days; 4) known SARS-CoV-2 exposure, excluding exposure at workplaces, in the previous 14 days; and 5) receipt of at least one dose of a SARS-CoV-2 vaccine. Each outcome was evaluated in the pooled study population, except for SARS-CoV-2 vaccination, which was only available in the Self-Testing Study.

We performed logistic regression with generalized linear mixed models to estimate the adjusted odds ratio (aOR) and 95% confidence interval (CI) for each outcome, with the business sector treated as a random intercept. Models included weakly informative priors to deal with quasi-complete separation of some fixed effects observed in previous studies (8). Models were adjusted for individual and business characteristics as well as the study (to determine differences in risk between studies), as appropriate. Confounders considered included age, sex, smoking, other health factors, ethnicity, income based on forward sortation area, recent travel outside Québec, exposure to someone with SARS-CoV-2 and business size. We repeated all analyses stratified by study, sex, ethnicity, income and business sector. If directions of effect for characteristics assessed differed significantly, we assessed effect modification using likelihood ratio tests of models with versus without interaction terms. Data were analyzed using R (version 4.2.2) using base packages or the blme (version 1.0–5) and BhGLM (version 1.1.0) packages.

### Ethical approval

The original studies were approved by the research ethics board of the Research Institute of the McGill University Health Centre (2021–7057 and MP-37-2022-7762), as was the present study (2023-9046). Given the nature of a secondary analysis of data for the present study, a waiver of informed consent was obtained.

## Results

**Figure 1:**
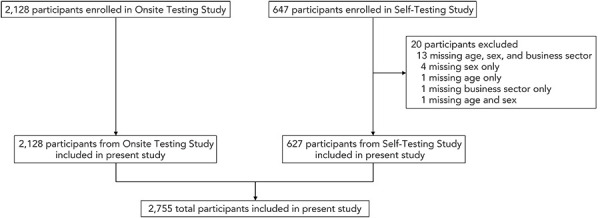
Number of participants enrolled in original studies that were ultimately included in the present analysis (n=2,755)

Overall, 2,775 participants completed a questionnaire between the two studies (Onsite Testing Study, n=2,128; Self-Testing Study, n=647), of which 2,755 were ultimately included in this analysis (**Figure 1**). All 20 exclusions pertained to the Self-Testing Study.

The median age of participants was 48 (IQR: 37–57) years, 1,154 (42%) were female, 1,527 (55%) identified as non-White and many (n=1,704; 62%) lived in areas with household incomes in the two lowest quintiles. Most participants (n=1,706; 62%) worked in the manufacturing/supplier sector and at businesses with more than 50 employees (n=1,755; 64%). Participant characteristics in terms of business sector and size, sex, self-reported ethnicity, and presence of health conditions varied significantly (*p*<0.05) between studies (**Table 1**). Disaggregated participant characteristics regarding ethnicity, income and business sector are in Appendix 2, Table S1.

**Table 1:** Characteristics of included participants in each study and overall

Characteristics	Number and percentage of participants in Onsite Testing Study N=2,128	Number and percentage of participants in Self-Testing Study N=627	Number and percentage of total participants N=2,755	*p*-value
Age, median (IQR), years	48 (IQR: 37–57)	48 (IQR: 34–57)	48 (IQR: 37–57)	0.477
**Business sector**				
Manufacturing/supplier	1,408 (66.2)	298 (47.5)	1,706 (61.9)	<0.001
Retail/customer facing	426 (20.0)	90 (14.3)	516 (18.7)	
Office	181 (8.5)	239 (38.1)	420 (15.2)	
Childcare	113 (5.3)	0 (0)	113 (4.1)	
**Business size**				
1–50 employees	895 (42.1)	105 (16.7)	1,000 (36.3)	<0.001
More than 50 employees	1,233 (57.9)	522 (83.2)	1,755 (63.7)	
**Sex**				
Male	1,320 (62.0)	281 (44.8)	1,601 (58.1)	<0.001
Female	808 (38.0)	346 (55.2)	1,154 (41.9)	
**Ethnicity**				
White	926 (43.5)	302 (48.2)	1,228 (44.6)	0.0441
Non-White	1,202 (56.5)	325 (51.8)	1,527 (55.4)	
**Income**				
Highest 60%	797 (37.4)	254 (40.5)	1,051 (38.1)	0.181
Lowest 40%	1,331 (62.5)	373 (59.5)	1,704 (61.8)	
**Any health factors**				
No	1,681 (79.0)	457 (72.9)	2,138 (77.6)	0.0015
Yes	447 (21.0)	170 (27.1)	617 (22.4)	
**Smoking history**				
Never smoked	1,668 (78.4)	484 (77.2)	2,152 (78.1)	0.563
Current/previous smoker	460 (21.6)	143 (22.8)	603 (21.9)	
**Recent travel outside of Montréal^a^**				
Yes	50 (2.3)	111 (17.7)	161 (5.8)	<0.001
No/not reported	2,078 (97.6)	516 (82.3)	2,594 (94.1)	
**Recent travel outside of Québec^a^**				
Yes	8 (0.4)	15 (2.4)	23 (0.8)	<0.001
No/not reported	2,120 (99.6)	612 (97.6)	2,732 (99.2)	
**Any contact with a person with confirmed COVID-19 outside of the workplace?**				
No	1,878 (88.2)	555 (88.5)	2,433 (88.3)	<0.001
Yes, previous (more than 14 days ago)	222 (10.4)	38 (6.1)	260 (9.4)	
Yes, casual recent (within 14 days)	28 (1.3)	34 (5.4)	62 (2.2)	
**Previous testing for SARS-CoV-2**				
No	1,155 (54.3)	138 (22.0)	1,293 (46.9)	<0.001
Yes	973 (45.7)	489 (78.0)	1,462 (53.1)	
Result was negative	882 (41.4)	450 (71.8)	1,332 (48.3)	0.438
Result was positive	91 (4.3)	39 (6.2)	130 (4.7)	
**SARS-CoV-2 vaccination^b^**				
No	N/A	68 (10.8)	N/A	N/A
Yes	N/A	559 (89.1)	N/A	
One dose	N/A	166 (26.5)	N/A	N/A
Two doses	N/A	393 (62.7)	N/A	

Abbreviations: IQR, interquartile range; N/A, not applicable

^a^ Recent travel defined as occurring within the previous 14 days

^b^ Information on vaccination only available for participants in the Self-Testing Study

Note: Two-sided *p*-values are calculated using Fisher exact or chi-squared test, as appropriate for categorical data and Wilcoxon rank sum or Kruskal-Wallis test for continuous data

Compared to participants in the Onsite Testing Study, those in the Self-Testing Study had significantly higher odds (78% vs. 46%; aOR 4.1, 95% CI: 3.2–5.2) of being previously tested for SARS-CoV-2 (**Table 2**). Moreover, participants with recent SARS-CoV-2 exposure had higher odds of previous SARS-CoV-2 testing compared to those without recent exposure (78% vs. 50%; aOR 4.0, 95% CI: 3.0–5.4). Older age (aOR 0.98, 95% CI: 0.98–0.99 per one-year increase) and being male (aOR 0.6, 95% CI: 0.5–0.7) were associated with lower odds. These findings regarding sex being associated with SARS-CoV-2 testing were largely consistent in stratified analysis (Appendix 2, **Tables S2 to S6**); however, we noted significant effect modification of sex by ethnicity, study and sector.

**Table 2:** Logistic regression results for characteristics associated with being tested for SARS-CoV-2 prior to study enrollment

Characteristics	Number and percentage of participants who received prior testing for SARS-CoV-2 (n/N)	aOR (95% CI)^a^
Age (per 1-year increase)	N/A	0.98 (0.98–0.99)
**Sex**		
Female	708/1,154 (61.3)	Ref.
Male	754/1,601 (47.1)	0.62 (0.52–0.73)
**Ethnicity**		
White	632/1,228 (51.5)	Ref.
Non-White	830/1,527 (54.3)	1.13 (0.94–1.36)
**Income**		
Highest 60%	554/1,051 (52.7)	Ref.
Lowest 40%	908/1,704 (53.3)	1.02 (0.86–1.21)
**Health factor**		
None reported	1,138/2,138 (53.2)	Ref.
Any reported	324/617 (52.5)	0.98 (0.80–1.21)
**Smoking history**		
Never smoked	1,146/2,152 (53.2)	Ref.
Current/previous smoker	316/603 (52.4)	1.11 (0.90–1.36)
**Any recent travel^b^**		
None reported	1,347/2,590 (52.0)	Ref.
Travel reported	115/165 (69.7)	1.15 (0.78–1.69)
**Any contact**		
None reported	1,209/2,433 (49.7)	Ref.
Contact reported	253/322 (78.6)	4.01 (3.01–5.35)
**Business size**		
1–50 employees	478/1,000 (47.8)	Ref.
More than 50 employees	984/1,755 (56.1)	1.00 (0.82–1.22)
**Study**		
Onsite Testing Study (January–March 2021)	973/2,128 (45.7)	Ref.
Self-Testing Study (July–October 2021)	489/627 (78.0)	4.10 (3.24–5.19)

Abbreviations: aOR, adjusted odds ratio; CI, confidence interval; N/A, not applicable; Ref., reference category

^a^ Business sector included as a random intercept in the model

^b^ Recent travel defined as occurring within the previous 14 days

Similarly, participants in the Self-Testing Study had higher odds of testing positive for SARS-CoV-2 more than four weeks prior to study enrolment (6.2% vs. 4.3%; aOR 1.7, 95% CI: 1.1–2.6) compared to those in the Onsite Testing Study (**Table 3**); there was no evidence (*p*=0.75) of effect modification by sector (Appendix 2, Table S6). We found those reporting recent SARS-CoV-2 exposure also had higher odds (aOR 3.9, 95% CI: 2.6–5.7) of testing positive. However, when limiting this analysis only to those who had previously been tested, there was no difference between studies in the odds of previously testing positive for SARS-CoV-2 (Appendix 2, **Table S7**). This was also the case in analyses limited to males (Appendix 2, Table S2) and those stratified on income (Appendix 2, Table S4).

**Table 3:** Logistic regression for characteristics associated with testing positive for SARS-CoV-2 more than four weeks prior to study enrollment

Characteristics	Number and percentage of participants who tested positive for SARS-CoV-2 more than four weeks prior to study enrollment (n/N)	aOR (95% CI)^a^
Age (per 1-year increase)	N/A	0.98 (0.97–1.00)
**Sex**		
Female	55/1,154 (4.8)	Ref.
Male	75/1,601 (4.7)	1.14 (0.77–1.70)
**Ethnicity**		
White	52/1,228 (4.2)	Ref.
Non-White	78/1,527 (5.1)	1.25 (0.82–1.88)
**Income**		
Highest 60%	46/1,051 (4.4)	Ref.
Lowest 40%	84/1,704 (4.9)	1.11 (0.76–1.62)
**Health factor**		
None reported	101/2,138 (4.7)	Ref.
Any reported	29/617 (4.7)	1.12 (0.72–1.75)
**Smoking history**		
Never smoked	107/2,152 (5.0)	Ref.
Current/previous smoker	23/603 (3.8)	0.78 (0.48-1.25)
**Any recent travel^b^**		
None reported	123/2,590 (4.7)	Ref.
Travel reported	7/165 (4.2)	0.72 (0.32–1.61)
**Any contact**		
None reported	87/2,433 (3.6)	Ref.
Contact reported	43/322 (13.3)	3.85 (2.60–5.71)
**Business size**		
1–50 employees	48/1,000 (4.8)	Ref.
More than 50 employees	82/1,755 (4.7)	0.95 (0.61–1.48)
**Study**		
Onsite Testing Study (January–March 2021)	91/2,128 (4.3)	Ref.
Self-Testing Study (July–October 2021)	39/627 (6.2)	1.65 (1.05–2.57)

Abbreviations: aOR, adjusted odds ratio; CI, confidence interval; N/A, not applicable; Ref., reference category

^a^ Business sector included as a random intercept in the model

^b^ Recent travel defined as occurring within the previous 14 days

When examining individual behaviours, participants in the Self-Testing Study had substantially higher odds (aOR 8.2, 95% CI: 5.6–12.1) of reporting recent travel outside Montréal or Québec (**Table 4**). Moreover, older participants (aOR 0.98, 95% CI: 0.97–0.99 per one-year increase) and those who identified as non-White (aOR 0.3, 95% CI: 0.2–0.5) had lower odds of reporting any recent travel. This was largely consistent in stratified analyses (Appendix 2, Tables S2 to S6), with evidence of effect modification (*p*<0.001) by business sector. When limiting this analysis to only those in the Self-Testing Study, vaccination was not associated with travel (Appendix 2, **Table S8**). We did not identify any difference between studies in terms of recent exposure to someone with confirmed SARS-CoV-2 in the total population (Appendix 2, **Table S9**), which was consistent in stratified analyses (Appendix 2, Tables S2 to S6).

**Table 4:** Logistic regression for characteristics associated with any self-reported travel outside of Montréal or Québec within the 14 days prior to study enrollment

Characteristics	Number and percentage of participants who reported any travel (n/N)	aOR (95% CI)^a^
Age (per 1-year increase)	N/A	0.98 (0.97–0.99)
**Sex**		
Female	62/1,154 (5.4)	Ref.
Male	103/1,601 (6.4)	1.38 (0.97–1.97)
**Ethnicity**		
White	127/1,228 (10.3)	Ref.
Non-White	38/1,527 (2.5)	0.30 (0.19–0.46)
**Health factor**		
None reported	123/2,138 (5.7)	Ref.
Any reported	42/617 (6.8)	1.18 (0.79–1.77)
**Income**		
Highest 60%	82/1,051 (7.8)	Ref.
Lowest 40%	83/1,704 (4.9)	0.83 (0.59–1.17)
**Study**		
Onsite Testing Study (January–March 2021)	50/2,128 (2.3)	Ref.
Self-Testing Study (July–October 2021)	115/627 (18.3)	8.24 (5.59–12.13)

Abbreviations: aOR, adjusted odds ratio; CI, confidence interval; N/A, not applicable; Ref., reference category

^a^ Business sector included as a random intercept in the model

Regarding SARS-CoV-2 vaccination (only available in the Self-Testing Study; **Table 5**) only participants from businesses with more than 50 employees had higher odds of receiving at least one vaccine dose (91% vs. 80%; aOR 2.6, 95% CI: 1.4–4.8). Findings were consistent in stratified analyses among males and those self-identifying as White (Appendix 2, Tables S2 to S3). In stratified analysis among those in the three highest income quintiles (Appendix 2, Table S4), participants identifying as non-White had lower odds of vaccination (aOR 0.3, 95% CI: 0.1–0.9), while participants working at businesses in the retail sector with more than 50 employees (Appendix 2, Table S6) had higher odds of vaccination (aOR 14.7, 95% CI: 3.5–61.1). We found significant effect modification on odds of vaccination by previous SARS-CoV-2 exposure and ethnicity, with those having previous exposure and self-identifying as White having significantly higher odds of vaccination compared to those self-identifying as non-White (Appendix 2, Table S3).

**Table 5:** Logistic regression for characteristics associated with receiving at least one dose of a vaccine against SARS-CoV-2 among the Self-Testing Study population after vaccine availability to this group

Characteristics	Number and percentage of participants who received one or more doses of a vaccine against SARS-CoV-2 (n/N)	aOR (95% CI)^a^
Age (per 1-year increase)	N/A	1.01 (0.99–1.03)
**Sex**		
Female	313/346 (90.5)	Ref.
Male	246/281 (87.5)	0.81 (0.48–1.38)
**Ethnicity**		
White	275/302 (91.0)	Ref.
Non-White	284/325 (87.4)	0.62 (0.34–1.14)
**Income**		
Highest 60%	233/254 (91.7)	Ref.
Lowest 40%	326/373 (87.4)	0.65 (0.37–1.13)
**Health factor**		
None reported	405/457 (88.6)	Ref.
Any reported	154/170 (90.6)	1.13 (0.62–2.06)
**Any recent travel^b^**		
None reported	453/512 (88.5)	Ref.
Travel reported	106/115 (92.2)	1.63 (0.78–3.44)
**Any contact**		
None reported	493/555 (88.8)	Ref.
Contact reported	66/72 (91.7)	1.58 (0.66–3.77)
**Business size**		
1–50 employees	84/105 (80.0)	Ref.
More than 50 employees	475/522 (91.0)	2.57 (1.39–4.75)

Abbreviations: aOR, adjusted odds ratio; CI, confidence interval; N/A, not applicable; Ref., reference category

^a^ Business sector included as a random intercept in the model

^b^ Recent travel defined as occurring within the previous 14 days

## Discussion

In this pooled analysis of prospective cross-sectional studies, we found significant increases in the number of non-healthcare essential workers being tested and testing positive for SARS-CoV-2 during 2021 in Montréal, with men the least likely to get tested. Mobility in the form of travel outside the Montréal area increased later in 2021. While the overall SARS-CoV-2 vaccination rate among non-healthcare essential workers was high, it was highest among those working at businesses with more than 50 employees.

Approximately four out of five participants reporting recent SARS-CoV-2 exposure received a PCR test, substantially more than people not reporting previous exposure, suggesting adherence with public health guidance and messaging surrounding SARS-CoV-2 testing. However, we did not find any difference in the proportion of participants reporting recent SARS-CoV-2 exposure between studies. This observation is likely driven by the variable intensity of public health measures and differing levels of virus circulation during each study period. Aggressive public health measures were in place during the Onsite Testing Study period, where approximately 1,000 people tested positive for SARS-CoV-2 daily in Québec (21). However, a subsequent reduction in measures during the Self-Testing Study, and attendant increases in travel outside the Montréal area observed in our analysis, likely did not increase the number of effective contacts, as 400 people tested positive each day during this period.

These data also provide insight into population behaviours. We found a higher likelihood among women than men for being previously tested for SARS-CoV-2, which aligns with literature on the higher frequency of healthcare access and of preventive health actions among women (22–26). Overall vaccine uptake among participants was high (89.1%) and equivalent to the proportion of adults 18 years of age or older who had received at least one COVID-19 vaccine dose in Québec by the end of the Self-Testing Study (27). However, vaccine uptake was highest among people working in businesses with at least 50 employees, which we speculate could be due to factors such as employer encouragement, availability of onsite vaccination efforts, motivation to prevent transmission in larger workplaces stemming from a sense of community (28,29) or business neighbourhood location within the Greater Montréal Area. We did not find any significant difference in SARS-CoV-2 vaccine uptake by self-reported ethnicity and neighbourhood income, though these factors were explored in a large survey of vaccine intent among Canadian adults (26). The survey found those with the lowest household income had higher odds of responding that they were unlikely to get vaccinated; however, racialized populations had lower odds of providing this response. Taken together, our analysis can be used to support decision-making and targeting of future public health programs to encourage preventive health behaviours, such as encouraging testing among men and simplifying access and/or encouraging vaccination among smaller businesses.

## Strengths and limitations

Key strengths of this study are the relatively large populations included in the analysis, the diverse nature of participants permitting exploration of various demographic and business-related factors, use of identical questionnaires for data collection, and temporal differences in data collection between studies, which allowed evaluations over time. This study is nonetheless subject to limitations. Some responses may have been impacted by recall biases as participants had to report previous testing history, travel and contact, though for most of these variables, the recall period was only 14 days. Only businesses experiencing a SARS-CoV-2 outbreak were included in the Self-Testing Study and larger businesses were preferentially recruited, which may have led to selection bias. Moreover, studies were conducted over different time periods in a rapidly evolving pandemic, and participant profiles between studies differed on demographic characteristics. We did not collect details on motivations for specific behaviours (e.g., decision to travel) and thus were unable to evaluate these aspects. Participant characteristics and behaviours were self-reported, which may have led some participants to not respond truthfully to some questions due to fear of consequences. We examined several outcomes in this study, and this may increase our risk of type I error.

## Conclusion

This pooled analysis of non-healthcare essential workers in Montréal in 2021 found that men were less likely to get tested for SARS-CoV-2 and those working at businesses with 50 or fewer employees were less likely to be vaccinated against SARS-CoV-2, but there were no significant differences in vaccination rates by sex, income or ethnicity. These data may contribute to decision-making regarding the design of testing and vaccination programs, as well as the allocation of resources to improve equity and the uptake and effectiveness of interventions for SARS-CoV-2 and other health threats (30).

## Appendix

Supplemental figures and tables are available upon request to the author at: jonathon.campbell@mcgill.ca

### Appendix 1: Participant questionnaires

Participant questionnaire from the Onsite Testing Study

Participant questionnaire from the Self-Testing Study

### Appendix 2: Data harmonization

Table S1: Characteristics of included participants and businesses in each study and overall

Table S2: All analyses stratified on sex

Table S3: All analyses stratified on self-reported ethnicity

Table S4: All analyses stratified on neighborhood income quintiles

Table S5: All analyses stratified on study

Table S6: All analyses stratified on business sector

Table S7: Logistic regression for characteristics associated with testing positive for SARS-CoV-2 more than four weeks prior to study enrollment among those who were tested

Table S8: Logistic regression for characteristics associated with any self-reported travel outside Montréal or Québec within the 14 days prior to study enrollment among those in the Self-Testing Study only

Table S9: Logistic regression for characteristics associated with any self-reported contact with someone with SARS-CoV-2 outside of the workplace within the 14 days prior to study enrollment
